# Psychological Impacts of Teaching Models on Ibero-American Educators during COVID-19

**DOI:** 10.3390/bs13120957

**Published:** 2023-11-21

**Authors:** Simone Nomie Sato, Emilia Condes Moreno, Adriana Rico Villanueva, Paulo Orquera Miranda, Pascual Chiarella, Gloria Bermudez, Jose Francisco Tornero Aguilera, Vicente Javier Clemente-Suárez

**Affiliations:** 1 Faculty of Sports Sciences, Universidad Europea de Madrid, 28670 Madrid, Spain; vctxente@yahoo.es; 2 Faculty of Biomedical and Health Sciences, Universidad Europea de Madrid, 28670 Madrid, Spain; 3 Centro de Opinión Pública, Universidad del Valle de México, Ciudad de México 72824, Mexico; 4 CMUCH, Centro Mexicano Universitario de Ciencias y Humanidades, Puebla 74240, Mexico; 5 Facultad de Ciencias de la Salud, Universidad Peruana de Ciencias Aplicadas, Lima 15023, Peru; 6 Escuela Colombiana de Rehabilitación, Bogotá 111711, Colombia; 7 Grupo de Investigación en Cultura, Educación y Sociedad, Universidad de la Costa, Barranquilla 080002, Colombia

**Keywords:** COVID-19, online teaching, Ibero-American, psychological impacts, hybrid teaching, mental health, professor

## Abstract

Educational systems globally, and notably in the Ibero-American context, underwent significant adaptations in response to the myriad challenges imposed by the COVID-19 pandemic. The pedagogical evolution unfolded through three discernible phases: predominantly online, hybrid, and ultimately, a return to face-to-face instruction. While these phases were universally apparent, cultural, socio-economic, and health disparities across regions subtly influenced the quality and experiential aspects of teaching and learning within these models. This study seeks to illuminate the psychological profiles and evaluative perspectives regarding teaching and learning quality among university educators during COVID-19’s tri-phase educational transformation. Engaging 601 university instructors from various Ibero-American countries, a comprehensive questionnaire mapped demographic, academic, and psychological landscapes across the pandemic’s distinctive epochs. The pivot to online educational methodologies, supplanting traditional modalities, permeated numerous facets of the educational endeavor, particularly impacting faculty life and wellbeing. Data underscored a prevalent sentiment of loneliness, indicative of broader mental health challenges, especially pronounced among educators in Latin American nations. Notwithstanding these hurdles, Latin American educators demonstrated a predilection towards online instruction, in stark contrast to their European peers, who exhibited a preference for in-person pedagogy. This study unveils the divergent pedagogical preferences and mental health challenges among university educators in the Ibero-American realm during COVID-19’s educational shifts, underlining the need for adaptable educational frameworks and robust mental health support, attuned to the region’s distinct socio-cultural and economic contexts.

## 1. Introduction

The appearance of the novel coronavirus (2019-nCoV) in December 2019 led to governments worldwide implementing strict measures in an effort to slow down its rapid spread. These measures included mandatory home quarantines [1,2,3]. On 30 January 2020, the World Health Organization (WHO) heightened the seriousness of the situation by declaring the virus a global public health emergency [2]. By 29 March, a range of measures, such as the sudden closure of public spaces and educational institutions, were put into effect [3]. As the number of confirmed cases and fatalities began to decline, public spaces gradually reopened, though with the requirement of wearing face masks being enforced [4]. The education sector, spanning primary through higher education, and impacting 1.3 billion learners globally, encountered profound disruptions, necessitating the implementation of rigorous protective measures [5,6,7].

Between 2020 and 2022, the educational landscape underwent a pivotal transformation, introducing an online instructional model anchored in virtual environments to sustain educational continuity. This shift introduced dual modalities: synchronized sessions, involving simultaneous engagement of instructors and students, and asynchronous sessions, where recorded lectures were made accessible to students, with practical sessions being delayed or substituted where feasible [1]. Particularly, in the initial six months, universities across Ibero-American countries largely transitioned to a wholly online format [8]. This adaptation, while successfully maintaining the pedagogical flow, unveiled a myriad of challenges including compromised teaching quality due to inadequate digital media planning, overburdened faculty, technological access disparities among students, and a deficiency in pedagogical support for instructors [9].

The imposition and evolution of online pedagogical methodologies have invariably impacted not only the caliber of instruction but also pervaded the quality of life and mental well-being of academic professionals. Teaching, inherently intricate, is recognized as one of the vocations most besieged by stress, exhibiting doubled instances of sadness and anxiety relative to other professions [10]. Professors have articulated challenges such as adaptation difficulties, a scarcity of virtual pedagogical resources, inexperience, constrained resources and time, and a sensed diminution in student engagement and discourse within online instructional environments [8,11,12]. A prevalent issue underscored by educators is the amplified temporal demand and the augmented challenge of sustaining students’ attention [13]. Consequently, it is pivotal to develop and implement pedagogical models that are not only adapted to the current educational climate but also aimed at equipping teachers with requisite skills, thereby alleviating the mental health impact and demanding workload.

Despite consistent socio-sanitary quarantine control procedures across all Ibero-American countries and uniform adoption of online instructional models, variances in cultural, economic, political, and contextual factors between nations significantly influenced the efficacy of online teaching and learning. The divergences between Latin American and European education systems in developing countries are notably pronounced, with a heightened focus on the structural impediments of emergency virtual education—such as access to computers and the internet, availability of conducive home study areas, and the dynamics of family-school relationships. Existing deficits in conventional modalities were accentuated by moments of disconnection, extending gaps and posing additional challenges for academics [14]. The lack of opportunities for continuous learning in technology, coupled with economic struggles—such as insufficient pay to procure essential equipment for personal and professional use—may have particularly impacted Latin American countries. Furthermore, the ramifications of the pandemic are intertwined with racial and income disparities, which create access gaps to crucial sanitary and health services [15].

Moreover, understanding academics’ perceptions of teaching quality from an Ibero-American vantage is pivotal. To achieve this, it is essential to explore three distinct phases: online instruction during the initial quarantine/lockdown, a hybrid phase during which access to public spaces, including educational institutions, was incrementally reinstated with capacity and sanitary restrictions, and finally, “the return to normality”, characterized by lifted restrictions and the resumption of in-person learning. Although these phases are well-differentiated in the literature, they transpired against the backdrop of varying COVID-19 wave timelines [16].

## 2. Materials and Methods

This research aims to elucidate the psychological profile and perceptions related to the quality of teaching-learning processes among university professors throughout the three stages (online, hybrid, and face-to-face) of COVID-19. Consequently, the initial hypothesis posits that European professors might perceive a higher teaching quality within the online model while experiencing reduced stress and loneliness compared to their Ibero-American counterparts. These suppositions align with prior research in this area and provide an initial approach to the actual aim [17].

To reach the study aim a study encompassing 601 university professors from Ibero-American countries, spanning ages 24 to 75, was carried out over a six-month period, from December 2021 to June 2022. The professors exhibited the following demographic and professional characteristics: mean age of 44.8 ± 10.5 years, BMI of 25.4 ± 4.5, with 44% males and 56% females. A substantial portion, 76%, were from Latin American nations (including Brazil, Peru, Colombia, and Mexico), while the remaining 24% were from European countries (Spain and Portugal). Faculty members from the Health Sciences constituted 52.6%, followed by Social Sciences at 22.3%, Architecture and Engineering at 7.5%, and other subjects comprising 17.7%. Additionally, 64.6% of professors indicated having moderate to extensive prior experience with online teaching, 57% affirmed proficiency in utilizing digital resources, and a remarkable 95.0% confirmed having access to necessary technological devices for online teaching, such as WIFI or a computer.

The study involved the administration of online questionnaires to conduct interviews with these professors. The inclusion criteria for participation were that the professors had to be actively teaching university courses throughout all phases of the pandemic, and they could come from any academic discipline.

To ensure that there were no duplicate responses from the same individual, professors were required to provide their ID, which was cross-checked against the university’s database. This research adhered to the Helsinki Declarations on human research and received authorization from the Ethics Committee of Universidad Europea de Madrid (CIPI/ 213006.55). All participants provided digital signatures indicating their agreement to participate, with the study’s objectives and methodology being clearly outlined.

The research was designed as a cross-sectional study, with an analysis of various parameters aimed at achieving its research objectives.

### 2.1. Demographic Information

This section of the study examined demographic variables, including gender, age (in years), country of residence, city of residence, environmental conditions during the lockdown, availability of digital resources for online classes, and the number of cohabitants.

### 2.2. Academic Information

In this section, the study gathered academic details from the participants. These details included research interests, the levels of education they taught (undergraduate, graduate, and postgraduate), the type of classes they conducted (synchronous or asynchronous), the availability of digital resources, the timing of classes during the pandemic, and whether or not classes were recorded.

### 2.3. Classes during the Pandemic Period

This section evaluated three distinct phases of education during the pandemic:

(a) Lockdown phase/online instruction: All classes shifted to emergency remote instruction, and learning occurred exclusively online;
(b) Hybrid phase: A combination of online and face-to-face instruction with reduced enrollment due to COVID-19 constraints;
(c) Presence phase/face-to-face: Return to in-person classes without capacity constraints but with COVID-19 restrictions.

In each phase, academics were asked to rate their stress levels, motivation, teaching effectiveness, convenience of teaching, workload, teaching challenges, and class format choice on a Likert scale ranging from 1 (lowest) to 5 (highest).

### 2.4. Psychological Factors

This section analyzed participants’ psychological profiles using:

(a) UCLA Loneliness Scale [18], which assessed feelings of disconnection from others on a Likert scale ranging from 1 (rarely) to 3 (frequently);
(b) STAI Scale: State-Trait Anxiety Inventory [19], which differentiated between “state anxiety” and “trait anxiety” on a Likert scale ranging from 1 (not at all) to 4 (very much);
(c) PSS-4: Perceived Stress Scale [20], consisting of four items measuring the degree to which life situations were perceived as stressful on a Likert scale ranging from 0 (never) to 4 (very often).

### 2.5. Statistical Analysis

Data analysis was performed using SPSS (version 21.0; SPSS, Inc., Chicago, IL, USA). To validate the normality of the data, the Kolmogorov–Smirnov test was applied. Differences between countries were analyzed using a *t*-test for independent samples, with the significance level set at *p* ≤ 0.05.

In pursuing a nuanced understanding of the professors’ adaptability and experiences amidst the pandemic-induced pedagogical shifts, a key determinant was their prior experience in online teaching environments. Recognizing the necessity for a methodical classification of professors based on their online teaching experience, we embarked on an extensive literature review to seek established frameworks or indices. However, the literature did not yield a classification scheme that resonated with the particular objectives and context of our study.

Given the absence of a pre-existing classification framework, we formulated an ad-hoc classification approach to categorize professors based on their prior experience in online environments. A composite variable was created by aggregating the scores from two questionnaire items: online teaching experience (rated on a scale of 1–10) and digital tools experience (rated on a scale of 1–10). This composite variable, with a potential range of 2–20, encapsulates a holistic view of the professors’ acumen and comfort in online teaching realms, amalgamating not only their direct teaching experience but also their proficiency with digital tools which are integral to online pedagogy.

To achieve a meaningful segregation of experience levels, participants were classified into three distinct categories using the 33% and 66% percentile analysis approach. This technique was adopted to ensure a balanced and statistically sound distribution of participants across the categories of low (9), medium (10–15), and high (>16) online experience levels. By dividing this variable into thirds, we aimed to create a robust yet flexible classification that allows for a discerning analysis based on professors’ prior online experience.

This ad-hoc classification, tailored to the investigative contours of our study, served as an instrumental axis for analyzing the interplay between prior online teaching experience and the professors’ evaluative perspectives and psychological profiles during the pandemic’s tri-phase educational transformation. Through this approach, we aspire to render a richer, contextually grounded analysis that could potentially inform future studies and contribute to the burgeoning discourse on online pedagogy in the face of unprecedented educational challenges.

## 3. Results

As illustrated in Table 1, there were notable regional disparities in the perceived teaching experiences during the lockdown period. Latin American professors consistently reported higher scores compared to their European counterparts across several metrics, including convenience in teaching, motivation to teach, preferred teaching methods, and teaching method preferences during both the lockdown and hybrid phases. Conversely, European professors scored higher in terms of teaching difficulty during the lockdown, stress levels during the hybrid phase, as well as teaching difficulty and highly demanding tasks during the hybrid phase. Furthermore, European professors displayed greater motivation to teach, perceived teaching quality, convenience in teaching, and a preference for teaching methods during the face-to-face phase.

Table 2 also shows differences between regions found regarding psychological factors on the UCLA—loneliness scale. Psychometric profiles suggest that Latin American professors have a profile marked by higher levels of loneliness.

In the study, 31% (*n* = 188) of the professors reported having high prior experience in online teaching and digital tools. Among these, 77% (*n* = 144) were professors from Latin America. This suggests that a significant proportion of this group had higher values (2.1 ± 0.8) when compared to European professors (1.8 ± 0.7) in terms of their prior digital teaching experience. The result was statistically significant (*p* < 0.05) with a 95% confidence interval ranging from −0.430 to −0.164, indicating that Latin American professors generally had higher prior digital teaching experience compared to their European counterparts.

Furthermore, when comparing the results of prior digital experience with the age of professors, the study found no statistically significant differences between professors with high and low levels of prior digital experience. This suggests that age did not play a significant role in determining the level of prior digital experience among the professors.

Overall, these findings indicate that Latin American professors had a higher level of prior digital teaching experience than European professors, and this difference was statistically significant. However, the study did not find a significant correlation between age and prior digital teaching experience among the professors.

## 4. Discussion

The primary objective of this study was to generate a comprehensive report on the mental well-being and the perception of teaching and learning experiences among university professors across three distinct educational scenarios during the COVID-19 pandemic: online, hybrid, and face-to-face settings, spanning both European and Latin American nations. Initially, our hypotheses posited that European academics would hold a more favorable view of the quality of online instruction and experience less stress and isolation compared to their Latin American counterparts. However, our hypothesis was not substantiated by the data. Instead, the study revealed that Latin American professors had a higher perception of teaching quality during the online phase, while European professors expressed a greater perception of teaching quality during the face-to-face phase. These unexpected findings underscore the complexity of the experiences and perceptions of university professors during the pandemic, with regional nuances playing a significant role in shaping their views on teaching and learning under different educational scenarios.

In exploring this dimension, it was discerned that Latin American professors exhibited a notably stronger preference for online teaching during the lockdown compared to their European counterparts, who conversely manifested significantly higher values related to teaching difficulty during the same period. Despite the implementation of stringent and precocious measures in Latin America, the effectiveness of these strategies was ostensibly constrained by pre-existing vulnerabilities within public health institutions, which were characterized by pervasive economic informality, suboptimal testing capabilities, and a palpable absence of a comprehensive contact tracing strategy [21]. These limitations curtailed the efficacy of the initiated measures and their capacity to staunchly curtail the proliferation of COVID-19, ultimately culminating in detrimental health and economic repercussions for these nations, epitomized by escalating unemployment rates and a substantive economic contraction [22]. These circumscribing circumstances and contextual milieu may have galvanized the proclivity among Latin American educators towards online instruction, potentially perceiving it as a modality that afforded a psychologically secure environment, within which the educational community could navigate the tribulations engendered by the COVID-19 epoch [22]. As a result, the growing preference for virtual learning environments, influenced by a combination of practical and psychological factors, represents a multi-dimensional response to the challenges brought about by the pandemic. This phenomenon warrants deeper investigation and a contextual understanding within the broader educational and socio-economic context.

Furthermore, resilience is conceptualized as the capability to adeptly navigate through adversity, wherein positive emotions play a pivotal role in confronting and mitigating challenging situations [23]. It is imperative to acknowledge that resilience is not an intrinsic attribute or one that is hereditarily transmitted through generations; rather, it materializes and matures throughout an individual’s lifespan, often sculpted by encounters with adverse events and the mastery of emotional self-regulation. Resilience is frequently associated with capabilities such as planning, learning, innovating, creating, and adaptive modifying [24], serving as a protective mechanism that individuals construct to adeptly navigate through stressful and formidable circumstances within their contextual environments.

In the context of Latin American countries, socio-natural disasters have persistently emerged as predominant impediments to developmental trajectories [25]. This has been exacerbated by the establishment of a neocolonial economic framework predicated on the extraction of communal resources, a perpetually escalating trajectory of urban poverty, stark social disparities, institutionalized racism, gender-based subjugation, environmental deterioration, and a conspicuous absence of risk-mitigated territorial planning [26,27]. In this vein, the social resilience that has been cultivated in these regions—borne out of a symbiosis between vulnerability and resilience—may elucidate the fortitude with which Latin American communities have contended with the catastrophic milieu of the pandemic. This also potentially explains the professors’ inclination towards a distance-learning model [9,28,29]. Contrastingly, European professors encountered more pronounced difficulties in teaching during the lockdown, a phenomenon consistent with preceding reviews [30]. This suggests that distinct socio-economic, cultural, and infrastructural contexts underpin the varied responses and adaptive strategies deployed by academic professionals across different geographic locales during the pandemic. Consequently, a nuanced understanding of these disparities is integral to formulating contextually pertinent and sustainable educational strategies in anticipation of future crises.

Moreover, according to the acquired data, European lecturers had higher levels of stress, teaching difficulty, and demanding activities during the hybrid period. This kind of instruction necessitates additional skills and effort on the part of professors to guarantee that the material reaches both in-class and at-home students. Moreover, this type of learning environment necessitates significant pedagogical adaptations to effectively leverage modern technologies. Previous studies conducted prior to the COVID-19 pandemic have emphasized that the hybrid learning setting demands greater coordination, enhanced skills, and additional teacher preparation [31]. Professors must be attentive to both physical and virtual locations, requiring them to perform specific actions on the teaching and learning platform. This added complexity translates into a heightened cognitive load for professors [32]. Additionally, when implementing synchronous hybrid learning, professors often encounter challenges in activating and engaging remote students to the same extent as those physically present in the class [33]. During the face-to-face phase, European academics exhibited greater motivation, a stronger drive to teach, higher perceived teaching quality, enhanced teaching convenience, and a preference for traditional teaching techniques [34]. This may be attributed to professors feeling more secure in traditional lectures, which offer immediate feedback, compared to online or hybrid format lessons [35]. Furthermore, professors underscore the significance of the campus experience for students, assigning more weight to face-to-face interactions with academics over technology [28]. They express resistance to online teaching, as it can lead to a sense of disembodied identities and disrupt their academic presence [36].

Conversely, within the context of the current study, a mere 32% of professors indicated having extensive online teaching experience prior to the pandemic, with 24% of these being from Ibero-American nations. This illustrates that Latin American professors possessed only a moderate familiarity with online education before the pandemic ensued, a finding that finds resonance with previous research [29]. Moreover, a retrospective examination of the digitization evolution in Latin American and Caribbean nations indicates that the pre-pandemic expansion of information technology (IT) in education was underscored by disruptive innovation, which enveloped the integration of innovative operational initiatives such as wearable technology, machine learning, and video games. Notably, instructors’ digital proficiency, training, experience, and attitudes toward technology play a significant role in influencing students’ learning journeys and their perceptions of virtual classroom quality [37].

During the lockdown, Latin American students expressed experiencing higher levels of loneliness compared to their European counterparts. Nonetheless, the values obtained from both cohorts are not therapeutically significant. As such, further research, more in-depth and encompassing, is necessitated to elucidate this topic with greater clarity and specificity. Pertinently, measures instituted to mitigate the spread of COVID-19, particularly those involving physical distancing, negatively impinged upon the mental health of the community by amplifying feelings of loneliness [38,39]. These phenomena delineate the intricate interplay between psychosocial experiences and educational modalities during pandemics, which warrants thorough exploration to devise holistic and supportive educational strategies in future instances of widespread crises.

Loneliness is associated with a range of detrimental psychological and organizational consequences, including decreased job performance, reduced work quality, lower motivation and commitment, diminished job satisfaction, increased intentions to leave one’s job, and a decline in overall well-being [40]. While loneliness has been examined in the context of education, it hasn’t been extensively explored concerning university professors in the aftermath of the COVID-19 pandemic. The well-being and performance of professors were adversely affected by the pandemic, particularly during the lockdown, as they often felt isolated in their work. Research indicates that interpersonal interactions played a significant role in determining the level of loneliness experienced by academics, influencing performance-related outcomes and overall quality of life during the pandemic. Additionally, working in an empty home environment can contribute to feelings of loneliness [41,42]. Therefore, evidence suggests that fostering supportive relationships in the workplace can serve as a vital protective factor against teacher loneliness and burnout [43]. Building and maintaining such relationships can play a crucial role in mitigating the negative effects of loneliness on educators’ well-being and job performance.

Certainly, previous studies have provided valuable insights into the multifaceted impacts of the COVID-19 pandemic on various aspects of the academic community. For instance, previous research emphasized the role of social media and anxiety in compliance with pandemic-related measures, providing insights into the intricate relationship between digital communication and individuals’ mental well-being during lockdowns [44]. Meanwhile, other authors delved into cultural differences among university students in terms of online learning quality and psychological profiles, further underlining the role of culture in adapting to remote education [45]. Additionally, a study explored the issue of misinformation during the health crisis, emphasizing the critical need for accurate information dissemination in the context of public health and education [46]. These studies collectively emphasize the holistic impact of the COVID-19 pandemic on students’ lives and the importance of considering various factors in the design of educational strategies during crises.

The transition to online and hybrid educational models has not only altered the pedagogical landscape but also blurred the boundaries between professional and personal domains for many educators. The exigencies of household circumstances, be it familial obligations, personal illnesses, or other COVID-19-related experiences, could significantly intersect with the professional realm, possibly exacerbating the challenges encountered in online instruction. For instance, professors navigating through the demands of childcare or eldercare amidst a global health crisis may find the online teaching landscape to be more taxing, thus potentially impacting their stress levels, engagement, and overall effectiveness. The differential impact of these external factors underscores the necessity for a more nuanced understanding of the myriad stressors that professors may contend with in the digital teaching paradigm. Incorporating an examination of these household and personal factors in future research could provide a more holistic understanding of the professors’ experiences, thereby contributing to the development of more supportive and adaptive educational strategies that cater to the diverse needs and circumstances of educators in a post-pandemic academic landscape. This enriched understanding could serve as a foundation for fostering a more conducive and empathetic educational environment, ultimately enhancing the quality and effectiveness of online instruction in the long term.

### 4.1. Practical Application

After several months of online experiences, an educational paradigm shift has occurred. The coronavirus crisis was a profound and unexpected shock, but it is not likely to be the last. After the COVID-19 outbreak, we have realized the need to use technology in the classroom. The execution of this educational concept, however, is fraught with obstacles. These challenges are associated with the innovative aspects of online education and its technological complications, teaching approaches, evaluation processes, student interaction, and faculty development. Universities must continue to invest in online education to improve the learning experience. Professors must receive proper training in digital skills, online approaches and tools, technological assistance, and enhanced student-teacher engagement.

### 4.2. Limitations of the Study and Future Research Lines

This study, while shedding light on some significant aspects of online instruction in the wake of a global pandemic, is subject to certain limitations. The reliance on self-reported data through questionnaires inherently introduces the potential for recall bias, considering the usage of memory-associated cognitive processes. However, the results gleaned remain pivotal, facilitating a comparative analysis between European and Latin American countries and elucidating the impact of the pandemic on the quality of online instruction. Looking toward future investigations, a more intricate exploration of each element of faculty life, scrutinized both singularly and with increased depth, and from variegated regional, national, or institutional viewpoints, could potentially unearth more context-specific implications. Moreover, a broader inquiry involving academic leaders in higher educational institutions might provide added insights into the perceived efficacy of online learning and the judiciousness of investments orchestrated during the pandemic epoch. An assessment that holistically examines the variegated impacts of online learning on students and educators alike, within the spectrum of their personal and professional domains, is also warranted to comprehend the overarching implications on both groups. It’s pertinent to note that the data presented here is of a preliminary nature, necessitating subsequent studies to affirm these findings with more comprehensive samples, ensuring the precision and generalizability of the conclusions drawn. Such progressive exploration will not only substantiate the findings of the present study but also pave the way towards developing educational strategies that are resilient, adaptive, and supportive in navigating future challenges and global crises.

Finally, at the time of conducting the study, we were only able to obtain the demographic data that had been provided. We acknowledge that the sample could have been more expansive and detailed, particularly with a larger trans sample. This limitation has been recognized and included in the revised manuscript under the section “Limitations of the Study”. We appreciate your valuable feedback and believe that addressing this limitation enriches the discourse surrounding our findings and contributes to the transparency and integrity of our research.

## 5. Conclusions

This study ventured into the exploration of the mental well-being and the perception of teaching and learning experiences among university professors across three different educational scenarios—online, hybrid, and face-to-face—during the COVID-19 pandemic, in both European and Latin American contexts. While our initial hypothesis assumed a more favorable perception of online instruction quality among European academics, the data highlighted a contrasting reality. Latin American professors exhibited a higher perception of teaching quality in online settings, perhaps finding a sense of psychological security in the virtual teaching landscape amidst the pandemic’s adversities. On the other hand, European professors resonated more with the quality of face-to-face instructional settings, reflecting a preference for traditional teaching modalities, possibly driven by the immediate feedback and interpersonal interactions inherent in such settings.

The nuanced preferences for different teaching modalities underline the intricate interplay of regional, socio-economic, and infrastructural contexts in shaping the academic experiences during these unprecedented times. The pandemic, in essence, accentuated the pre-existing vulnerabilities and showcased the disparities in digital readiness and adaptability between the two regions. The inclination towards online teaching among Latin American professors could be seen as a multi-dimensional response to the pandemic’s challenges, integrating both practical and psychological facets in navigating the crisis. Meanwhile, the European professors’ struggles during the online and hybrid phases reflect the need for substantial pedagogical adaptations and enhanced digital proficiency to effectively navigate the hybrid teaching landscape.

Moreover, the observations regarding professors’ mental well-being, particularly the feelings of loneliness and the consequential impact on job performance and satisfaction, underscore the necessity for fostering supportive workplace relationships and communities. The pandemic has not only disrupted the traditional educational paradigms but also propelled a global discourse on the psychological wellness of the academic community, the digital transformation of education, and the requisite strategies to ensure a resilient and adaptive educational ecosystem. The varied regional responses to the pandemic’s educational challenges, as unveiled in this study, offer a fertile ground for further investigations. Future research endeavors may delve deeper into the cultural, infrastructural, and socio-political determinants of these observed disparities, aiming to orchestrate educational strategies that are cognizant of and responsive to the diverse regional contexts and the evolving global challenges. Such nuanced understanding is imperative for crafting sustainable, resilient, and inclusive educational frameworks, poised to navigate the intricacies of the present and future global crises adeptly.

## Figures and Tables

**Table 1:** Regional differences in the perception of academic quality during lockdown.

Variables	Europe	Latin America	t	*p*	95% Confidence Interval	
					Lower	Upper
Age (years)	44.2 ± 10.1	45.3 ± 10.8	−1.247	0.213	−2.840	0.635
General stress level during lockdown (1–10)	6.2 ± 2.6	6.5 ± 2.7	−1092	0.275	−0.685	0.196
Motivation during lockdown (1–5)	3.4 ± 1.2	3.5 ± 1.1	−0.575	0.565	−0.244	0.133
Stress level during lockdown (1–5)	3.3 ± 1.3	3.2 ± 1.3	0.153	0.878	−0.197	0.231
Perceived teaching during lockdown (1–5)	2.5 ± 1.0	3.4 ± 1.1	−11063	0.000	−1163	−0.812
Convenience to teach during lockdown (1–5)	2.9 ± 1.3	3.8 ± 1.1	−8299	0.000	−1028	−0.635
Motivation to teach during lockdown (1–5)	2.9 ± 1.2	3.6 ± 1.1	−7473	0.000	−0.920	−0.537
Difficulty to teach during lockdown (1–5)	3.1 ± 1.3	2.7 ± 1.2	4006	0.000	0.211	0.618
Demanding activities during lockdown (1–5)	3.9 ± 1.1	3.9 ± 1.1	−0.180	0.858	−0.196	0.163
Preferred teaching method during lockdown (1–5)	2.0 ± 1.1	3.2 ± 1.4	−11203	0.000	−1400	−0.982
Stress level during the hybrid phase (1–5)	3.0 ± 1.3	2.8 ± 1.5	2243	0.025	0.034	0.514
Motivation during the hybrid phase (1–5)	3.3 ± 1.3	3.1 ± 1.5	1923	0.055	−0.005	0.461
Perceived teaching during the hybrid phase (1–5)	2.7 ± 1.0	2.8 ± 1.5	−1698	0.090	−0.423	0.031
Convenience to teach during the hybrid phase (1–5)	2.8 ± 1.2	3.0 ± 1.5	−1502	0.134	−0.415	0.055
Motivation to teach during the hybrid phase (1–5)	2.9 ± 1.2	3.0 ± 1.6	−0.559	0.576	−0.309	0.172
Difficulty to teach during the hybrid phase (1–5)	2.9 ± 1.3	2.4 ± 1.5	4273	0.000	0.279	0.753
Demanding activities during hybrid phase (1–5)	3.5 ± 1.3	3.2 ± 1.8	2258	0.024	0.040	0.575
Preferred teaching method during the hybrid phase (1–5)	2.1 ± 1.2	2.5 ± 1.6	−3486	0.001	−0.661	−0.185
Motivation during the face-to-face phase (1–5)	3.6 ± 1.6	3.1 ± 1.8	3293	0.001	0.195	0.772
Stress level during the face-to-face phase phase (1–5)	2.7 ± 1.3	2.7 ± 1.7	0.189	0.850	−0.228	0.276
Perceived teaching during the face-to-face phase (1–5)	4.0 ± 1.1	3.3 ± 1.8	5314	0.000	0.439	0.954
Convenience to teach during the face-to-face phase (1–5)	3.7 ± 1.4	3.1 ± 1.8	4336	0.000	0.330	0.876
Motivation to teach during the face-to-face phase (1–5)	3.7 ± 1.4	3.2 ± 1.8	4256	0.000	0.319	0.865
Difficulty to teach during the face-to-face phase (1–5)	2.3 ± 1.2	2.2 ± 1.5	0.756	0.450	−0.145	0.326
Demanding activities during the face-to-face phase (1–5)	2.9 ± 1.2	2.9 ± 1.8	0.067	0.947	−0.249	0.266
Preferred teaching method during the face-to-face phase (1–5)	3.8 ± 1.5	2.8 ± 1.9	6619	0.000	0.676	1246
General preferred teaching method (1–5)	1.5 ± 1.0	1.7 ± 1.2	−1817	0.070	−0.355	0.014

**Table 2:** Differences between regions of the psychological profile of students.

Variables	Europe	Latin America	t	*p*	95% Confidence Interval	
					Lower	Upper
STAI (1–4)	12.2 ± 3.8	12.1 ± 4.4	0.217	0.829	−0.61502	0.76746
UCLA (1–3)	4.3 ± 1.6	4.6 ± 1.8	−2.331	0.020	−0.62306	−0.05319
PSS-4 (0–4)	5.1 ± 3.2	5.3 ± 3.3	−0.605	0.546	−0.70118	0.37111

(STAI) State-Trait Anxiety Inventory scale; (PSS-4) Perceived Stress Scale; (UCLA) Loneliness Scale. Differences between genders (*p* < 0.05).

## Data Availability

All the data is presented in the study.

## References

[1] Farooq R.K., Rehman S.U., Ashiq M., Siddique N., Ahmad S. (2021). Bibliometric analysis of coronavirus disease (COVID-19) literature published in Web of Science 2019–2020. J. Fam. Community Med..

[2] Kannan S., Shaik Syed Ali P., Sheeza A., Hemalatha K. (2020). COVID-19 (Novel Coronavirus 2019)-recent trends. Eur. Rev. Med. Pharmacol. Sci..

[3] Tornero-Aguilera J.F., Rubio-Zarapuz A., Clemente-Suárez V.J. (2021). Implications of surgical mask use in physical education lessons. Physiol. Behav..

[4] Pollard C.A., Morran M.P., Nestor-Kalinoski A.L. (2020). The COVID-19 pandemic: A global health crisis. Physiol. Genom..

[5] Agrawal A., Bhardwaj R. (2020). Reducing chances of COVID-19 infection by a cough cloud in a closed space. Phys. Fluids.

[6] Güner H.R., Hasanoğlu İ., Aktaş F. (2020). COVID-19: Prevention and control measures in community. Turk. J. Med. Sci..

[7] Amato A., Caggiano M., Amato M., Moccia G., Capunzo M., De Caro F. (2020). Infection control in dental practice during the COVID-19 pandemic. Int. J. Environ. Res. Public Health.

[8] Azorín C. (2020). Beyond COVID-19 supernova. Is another education coming?. J. Prof. Cap. Community.

[9] Zhao Y. (2020). COVID-19 as a catalyst for educational change. Prospects.

[10] Santos G.M.R.F., dos Silva M.E., do Rego Belmonte B. (2021). COVID-19: Emergency remote teaching and university professors’ mental health. Rev. Bras. Saúde Matern. Infant..

[11] Stanistreet P., Elfert M., Atchoarena D. (2020). Education in the age of COVID-19: Understanding the consequences. Int. Rev. Educ..

[12] Pedró F. (2020). COVID-19 y educación superior en América Latina y el Caribe: Efectos, impactos y recomendaciones políticas. Análisis Carol..

[13] Santuario A.A. (2020). Educación Superior y COVID-19: Una Perspectiva Comparada.

[14] Teräs M., Suoranta J., Teräs H., Curcher M. (2020). Post-Covid-19 education and education technology ‘solutionism’: A seller’s market. Postdigital Sci. Educ..

[15] Qureshi S. (2021). Pandemics within the pandemic: Confronting socio-economic inequities in a datafied world. Inf. Technol. Dev..

[16] Nomie-Sato S., Moreno E.C., Villanueva A.R., Chiarella P., Tornero-Aguilera J.F., Beltrán-Velasco A.I., Clemente-Suárez V.J. (2022). Gender Differences of University Students in the Online Teaching Quality and Psychological Profile during the COVID-19 Pandemic. Int. J. Environ. Res. Public Health.

[17] Chan R.Y., Bista K., Allen R.M. (2021). Is Online and Distance Learning the Future in Global Higher Education? The Faculty Perspectives during COVID-19. Online Teaching and Learning in Higher Education during COVID-19.

[18] Russell D.W. (1996). UCLA Loneliness Scale (Version 3): Reliability, validity, and factor structure. J. Personal. Assess..

[19] van Knippenberg F.C., Duivenvoorden H.J., Bonke B., Passchier J. (1990). Shortening the State-Trait Anxiety Inventory. J. Clin. Epidemiol..

[20] Warttig S.L., Forshaw M.J., South J., White A.K. (2013). New, normative, English-sample data for the short form perceived stress scale (PSS-4). J. Health Psychol..

[21] Marinoni G., Van’t Land H., Jensen T. (2020). The Impact of Covid-19 on Higher Education Around the World.

[22] Buss P.M., Fonseca L.E. (2020). Cadernos CRIS-Fiocruz: Panorama da Resposta Global à COVID-19-Informe 19-Setembro/Outubro-2020.

[23] Martínez-Martí M.L., Ruch W. (2017). Character strengths predict resilience over and above positive affect, self-efficacy, optimism, social support, self-esteem, and life satisfaction. J. Posit. Psychol..

[24] Martínez-Ramón J.P., Morales-Rodríguez F.M., Pérez-López S. (2021). Burnout, resilience, and COVID-19 among teachers: Predictive capacity of an artificial neural network. Appl. Sci..

[25] Allen J., Rowan L., Singh P. (2020). Teaching and teacher education in the time of COVID-19. Asia-Pac. J. Teach. Educ..

[26] Montero-Hernandez V., Levin J., Diaz-Castillo M. (2014). Academic resilience and achievement: Self-motivational resources that guide faculty participation in instructional technology training at a Mexican university. J. Hisp. High. Educ..

[27] Sandoval-Díaz J. (2020). Vulnerabilidad-resiliencia ante el proceso de riesgo-desastre: Un análisis desde la ecología política. Polis. Rev. Latinoam..

[28] Chandasiri O. (2020). The COVID-19: Impact on education. J. Asian Afr. Soc. Sci. Humanit..

[29] Medina-Guillen L.F., Quintanilla-Ferrufino G.J., Palma-Vallejo M., Medina Guillen M.F. (2021). Workload in a group of Latin American teachers during the COVID-19 pandemic. Uniciencia.

[30] Thomas M.S., Rogers C. (2020). Education, the science of learning, and the COVID-19 crisis. Prospects.

[31] Ørngreen R., Levinsen K., Jelsbak V., Moller K.L., Bendsen T. (2015). Simultaneous class-based and live video streamed teaching: Experiences and derived principles from the bachelor programme in biomedical laboratory analysis. Proceedings of the 14th European Conference on E-Learning (ECEL 2015).

[32] Zydney J.M., McKimmy P., Lindberg R., Schmidt M. (2019). Here or there instruction: Lessons learned in implementing innovative approaches to blended synchronous learning. TechTrends.

[33] Weitze C.L. (2015). Pedagogical innovation in teacher teams: An organisational learning design model for continuous competence development. Proceedings of the ECEL 2015: The 14th European Conference on E-Learning.

[34] Adedoyin O.B., Soykan E. (2020). Covid-19 pandemic and online learning: The challenges and opportunities. Interact. Learn. Environ..

[35] Mielgo-Conde I., Seijas-Santos S., Grande-de-Prado M. (2021). Review about online educational guidance during the COVID-19 pandemic. Educ. Sci..

[36] El-Soussi A. (2022). The shift from face-to-face to online teaching due to COVID-19: Its impact on higher education faculty’s professional identity. Int. J. Educ. Res. Open.

[37] Rajab M.H., Gazal A.M., Alkattan K. (2020). Challenges to online medical education during the COVID-19 pandemic. Cureus.

[38] Dahlberg L. (2021). Loneliness during the COVID-19 pandemic. Aging Ment. Health.

[39] Martín-Rodríguez A., Tornero-Aguilera J.F., López-Pérez P.J., Clemente-Suárez V.J. (2022). The Effect of Loneliness in Psychological and Behavioral Profile among High School Students in Spain. Sustainability.

[40] Dor-Haim P., Oplatka I. (2021). Feelings of loneliness among school principals: Experiences, causes and copying strategies. Leadersh. Policy Sch..

[41] Adnan M., Anwar K. (2020). Online Learning amid the COVID-19 Pandemic: Students’ Perspectives. Online Submiss..

[42] Noor S., Isa F.M., Mazhar F.F. (2020). Online teaching practices during the COVID-19 pandemic. Educ. Process Int. J..

[43] Khalili H. (2020). Online interprofessional education during and post the COVID-19 pandemic: A commentary. J. Interprof. Care.

[44] Rodriguez-Besteiro S., Beltran-Velasco A.I., Tornero-Aguilera J.F., Martínez-González M.B., Navarro-Jiménez E., Yáñez-Sepúlveda R., Clemente-Suárez V.J. (2023). Social media, anxiety and COVID-19 lockdown measurement compliance. Int. J. Environ. Res. Public Health.

[45] Sato S.N., Condes Moreno E., Rico Villanueva A., Orquera Miranda P., Chiarella P., Tornero-Aguilera J.F., Clemente-Suárez V.J. (2022). Cultural Differences between University Students in Online Learning Quality and Psychological Profile during COVID-19. J. Risk Financ. Manag..

[46] Clemente-Suárez V.J., Navarro-Jiménez E., Simón-Sanjurjo J.A., Beltran-Velasco A.I., Laborde-Cárdenas C.C., Benitez-Agudelo J.C., Bustamante-Sánchez Á., Tornero-Aguilera J.F. (2022). Mis–dis information in COVID-19 health crisis: A Narrative review. Int. J. Environ. Res. Public Health..

